# Predictors of Rehabilitation Outcomes Following Pediatric Cochlear Implantation

**DOI:** 10.3390/jcm15030981

**Published:** 2026-01-26

**Authors:** Ke Wang, Zhihan Lin, Meiling Yan, Yan Rui, Haihong Liu

**Affiliations:** Department of Otolaryngology, Head and Neck Surgery, Beijing Children’s Hospital, Capital Medical University, National Center for Children’s Health, No. 56, South Lishi Road, Xicheng District, Beijing 100045, China; wangkeinwangyi@163.com (K.W.);

**Keywords:** cochlear implantation, hearing loss, pediatric, outcomes, predictors

## Abstract

Cochlear implantation (CI) is a well-established intervention for improving auditory and speech development in children with severe-to-profound hearing loss. Nonetheless, postoperative rehabilitation outcomes exhibit substantial individual variability. This review synthesizes contemporary evidence on predictors of rehabilitation success following pediatric CI. A robust set of general factors is consistently linked to more favorable outcomes, including earlier age at implantation (with particular benefit within the first year of life), stronger preoperative receptive language skills and speech recognition, higher developmental quotient and nonverbal intelligence, and higher parental educational level. Regarding hearing-specific variables, later-onset deafness, a shorter duration of deafness, and identifiable etiologies (notably specific genetic mutations such as GJB2 and OTOF) exert significant influence. Furthermore, bilateral CI demonstrates superior outcomes compared to unilateral CI, with the surgical timing (simultaneous versus sequential) and factors such as electrode array selection and placement being critical determinants. Overall, postoperative outcomes arise from a complex interplay of biological, developmental, and environmental factors.

## 1. Introduction

Hearing loss is one of the most prevalent congenital anomalies and the leading type of disability worldwide. Currently, disabling hearing loss—defined as a hearing threshold exceeding 30 dB HL in the better ear—affects an estimated 34 million children globally [1]. Early identification and timely intervention are critical in pediatric hearing loss. Without prompt management, it can severely impair auditory, speech, cognitive, and psychosocial development, leading to substantial personal, familial, and societal burdens [2,3,4]. Sensorineural hearing loss (SNHL) is the most prevalent form of permanent hearing impairment in children [5]. In cases of mild to moderate SNHL, rehabilitation is primarily supported by the use of hearing aids (HAs). For children with bilateral severe to profound SNHL or those who are unable to achieve functional auditory-verbal communication despite appropriate hearing aid amplification, cochlear implantation (CI) represents the most established and effective intervention. During the critical period of children’s brain development, experience-driven plasticity accelerates the maturation of auditory and language-related regions in the left hemisphere, leading to a specialized “left-lateralized” functional advantage for processing complex temporal and phonological information [6]. Sensory deprivation, particularly congenital hearing loss, directly disrupts this finely tuned developmental process. This disruption not only results in delayed maturation and maladaptive reorganization of the primary auditory cortex but also impairs the typical left-hemisphere dominance for language processing. Early provision of auditory access—through interventions such as HAs or CI—is therefore essential to support the normative development of language and higher cognitive functions in infants with hearing impairment [7].

However, significant inter-individual variability in CI outcomes is commonly observed among children with hearing loss in clinical practice. This heterogeneity has been documented across various etiological and clinical subgroups, including congenital versus acquired hearing loss [8], prelingual deafness versus postlingual deafness [9,10], and unilateral versus bilateral implantation [11,12]. Notably, children with auditory neuropathy spectrum disorder (ANSD), cochlear nerve deficiency (CND), inner ear malformations (IEMs), or additional comorbid conditions—collectively affecting approximately 40% of pediatric CI recipients—exhibit particularly unpredictable outcomes, with some deriving minimal or no functional benefit from the intervention [13,14]. Therefore, accurate prediction of postoperative rehabilitation outcomes is essential for tailoring intervention strategies and optimizing the allocation of medical resources. To date, there remains no consensus on a comprehensive set of prognostic factors or clearly defined high-risk populations who may experience limited postoperative gains. Current clinical prediction tools are often fragmented and lack sufficient predictive power. This review synthesizes existing evidence on predictive factors associated with CI outcomes in children, aiming to consolidate research findings and inform evidence-based clinical decision-making.

## 2. General Predictors

### 2.1. Age at Implantation

Implantation age remains one of the most robustly supported and strongly predictive clinical prognostic indicators currently available. It reflects the timing of intervention during the critical period of auditory-language neural plasticity in children. Extensive research has consistently identified specific age cut-off points with clear clinical implications, establishing a foundational framework for guiding decisions on early intervention. Research indicates that the human central auditory system exhibits a critical period of approximately 3.5 years for maximal neural plasticity, beyond which plasticity declines markedly, with a significant reduction observed after age 7 [15].

For children with bilateral severe to profound SNHL, the current consensus widely recommends CI before the age of 12 months [16]. Extensive evidence demonstrates that early implantation, particularly before 12 months, provides infants with the highest likelihood of achieving language development, speech production, and speech recognition abilities that approach or even parallel those of age-matched normal-hearing peers. Karltorp et al. [17] further showed that children implanted between 5 and 11 months attain age-appropriate language comprehension earlier and exhibit more favorable vocabulary development than those implanted between 12 and 29 months. According to the systematic review by Wu et al. [18], children who received CI at ≤12 months demonstrated vocabulary development after 10 years that most closely matched that of their normal-hearing peers in terms of language age. Those implanted between 12 and 24 months followed, while children implanted between 24 and 36 months showed the most substantial vocabulary gap. Furthermore, at both 5 and 10 years post-implantation, grammatical development in the ≤12-month cohort was closest to normal-hearing children, whereas outcomes for children implanted after 12 months consistently fell below the 75th percentile [18]. A prospective multicenter study by Dettma et al. [19] demonstrated that the 6–12-month-old CI group had significantly better speech production and comprehensive language standard scores than the older group, and more children in this cohort reached normal language levels at school entry.

In 2020, the U.S. Food and Drug Administration expanded the CI indication to children with bilateral profound SNHL aged ≥ 9 months [20]. Research shows that children who received CI activation before 9 months of age had Functional Listening Index scores close to those of normal-hearing children, while those activated between 9 and 24 months did not reach this level [20]. Additionally, another study showed that the pre-9-month CI group demonstrated significant improvement in receptive language starting 3 months after surgery, reaching the level of normal-hearing children of the same age by 9 months after surgery and maintaining this level until 2 years old [21].

### 2.2. Preoperative Receptive Language Skills, Speech Recognition and Speech Intelligibility

Preoperative language-related abilities and speech recognition are critical determinants of postoperative outcomes in late-implanted prelingually deaf individuals undergoing CI. The 2019 American Academy of Audiology’s Clinical Practice Guidelines for CI indicate that children and adolescents over 6 years of age should possess an established auditory and language foundation prior to CI [22]. Research has demonstrated that morphosyntactic comprehension ability serves as an independent predictor of open-set speech perception performance in pediatric CI users [23]. Furthermore, preoperative speech recognition capacity exhibits a strong correlation with postoperative outcomes and can function as a reliable prognostic indicator for early-deafened adolescents and adults following CI [24]. Haruo Yoshida et al. [25] reported that among 34 adolescents with prelingual or perilingual severe-to-profound hearing loss who underwent CI at age ≥ 10 years, postoperative Speech Discrimination Scores (SDSs) were significantly associated with both preoperative SDSs and preoperative hearing thresholds in the non-implanted ear. When late-implanted prelingually deaf children have acquired foundational language skills before implantation, they tend to achieve better auditory and speech rehabilitation outcomes. This advantage may stem from the brain’s ability to more efficiently integrate electrical input from the CI with pre-existing linguistic knowledge and auditory representations, facilitating faster sound adaptation and language comprehension. In contrast, those lacking prior language exposure require longer and more intensive, systematic rehabilitation to develop essential language competencies after surgery.

In early-implanted children with prelingual deafness, substantial evidence indicates that preoperative receptive language ability significantly influences postoperative language outcomes. Mitchell et al. [26] reported that higher preoperative scores on the Preschool Language Scales–Auditory Comprehension (PLS-AC) and younger age at implantation were significantly associated with improved PLS-AC performance following CI. Irina Castellanos et al. [27] further found that early preschool measures of receptive vocabulary and speech intelligibility predicted long-term speech and language development in pediatric CI users. In early-implanted children with prelingual deafness, early receptive language skills and speech intelligibility serve not only as key predictors of short-term postoperative language outcomes but also play a foundational role in shaping and reinforcing the neural architecture of the auditory-language pathway. This neurodevelopmental process may establish a critical substrate for the subsequent acquisition of higher-order language skills, such as grammar and syntax, thereby exerting a sustained and far-reaching impact on long-term speech and language development.

### 2.3. Developmental Quotient and Nonverbal Intelligence

Nonverbal intelligence refers to an individual’s ability to solve problems and understand the world by using visual, spatial, logical reasoning and other abilities without relying on language. Developmental quotient is a quantitative measure used to assess children’s neurodevelopmental levels and can be subdivided into several domains, including gross motor, fine motor, adaptive behavior, language, and personal social behavior [28]. Developmental quotient and nonverbal intelligence are strongly associated with hearing and speech outcomes, as well as expressive/receptive language skills in children with CI [29]. In terms of postoperative auditory and speech performance, Jiong Dang et al. [30] demonstrated that the Infant-Toddler Meaningful Auditory Integration Scale (IT-MAIS), Meaningful Use of Speech Scale (MUSS), Categories of Auditory Performance (CAP), and SIR scores of children (excluding those with developmental delay and autism spectrum disorder) two years after surgery were significantly positively correlated with the eye-hand coordination developmental quotient (DQ) and operational DQ, indicating that preoperative non-verbal intelligence development could predict postoperative outcomes to a certain extent. Yang Y et al. [31] confirmed that the adaptive ability DQ in the Gesell scale was positively correlated with the improvement of CAP/SIR scores one year after CI, while age was negatively correlated; the combination of adaptive DQ and age had good sensitivity and specificity in predicting the effect of CI in children, and the higher the preoperative adaptive DQ, the greater the possibility of good postoperative performance. Additionally, research has demonstrated that nonverbal intelligence quotient is a critical predictor of spectral modulation detection ability in children with CI, and this foundational auditory processing capacity, in turn, significantly predicts their segmental and suprasegmental speech perception [32]. The finding strengthens the role of nonverbal intelligence as a core predictor by linking it to underlying basic auditory processing mechanisms.

Regarding receptive and expressive language outcomes, the Geers team demonstrated that the Wechsler Intelligence Scale for Children-Third Edition (WISC-III) performance index, which is a key measure of nonverbal intelligence, can predict expressive language abilities in CI recipients [33]. Nonverbal intelligence accounted for 10% of the variance in receptive language ability and was associated with vocabulary size and parental education level [34]. Research demonstrates that non-verbal intelligence is a key predictor of reading ability development in children with CI, independently accounting for significant variance in both reading decoding and comprehension skills [35]. Dawson et al. [36] further showed that visual-spatial memory significantly predicted receptive language performance in a sample of 24 school-aged children with hearing loss.

### 2.4. Parental Educational Level

Parental educational level is widely regarded as a key family environmental factor for predicting rehabilitation outcomes in children with CI. Higher parental education is generally associated with greater cognitive and social resources, which may facilitate earlier access to implantation surgery and enhance the conditions necessary for postoperative auditory and speech development [16,37]. Lin et al. [38] found that parents with higher education levels possess more rehabilitation-related knowledge, which, in turn, positively predicts children’s auditory-speech outcomes after implantation. A systematic review and meta-analysis further support this, indicating that parental education is a significant predictor of postoperative language development, with a standardized regression coefficient of 0.45 (95% CI: 0.29–0.62), reflecting a moderate to strong association [39]. Importantly, Lee et al. [40] demonstrated that even when surgical and rehabilitation costs are fully covered by third-party funding—thereby controlling for financial barriers—parental education, especially maternal education, continues to significantly influence both earlier implantation age and better language outcomes. This highlights the role of non-economic pathways, such as cognitive stimulation and parenting practices within the home environment, in shaping rehabilitation success. However, not all studies align with this perspective. One single-center prospective study reported no statistically significant relationship between parental education and children’s auditory performance one year after implantation, as assessed by CAP scores [l]. The authors noted that contextual factors, including limited healthcare infrastructure such as the absence of an insurance system, may restrict the generalizability of their findings [41].

Overall, the evidence regarding the impact of parental education on postoperative rehabilitation remains inconsistent. While some studies point to benefits mediated through knowledge, resources, and timely intervention, others suggest that its role may be attenuated in specific healthcare and sociocultural settings. Future research utilizing larger, multi-center cohorts and longitudinal designs is needed to clarify whether parental education holds independent predictive value and to elucidate the specific mechanisms through which it may influence outcomes.

## 3. Predictors Related to Hearing Loss

### 3.1. Onset of Deafness and Duration of Deafness

The onset of deafness and the duration of deafness (DoD) are key determinants of CI outcomes in children. Later onset [42] and shorter DoD [43] are consistently associated with better postoperative recovery of auditory and speech abilities. In prelingually deaf children, the timing of hearing loss defines the critical window during which the auditory system is exposed to acoustic input. Early-onset deafness not only triggers cross-modal reorganization of the auditory cortex and degeneration of spiral ganglion neurons [44,45], but may also disrupt the typical left-hemisphere lateralization of the language network. Evidence indicates that deprivation of early auditory experience impairs both the functional maturation and hemispheric specialization of the left-hemisphere language system [7], thereby compromising neural efficiency and behavioral outcomes in language acquisition [6]. Thus, early implantation serves not merely to restore auditory input, but crucially to support the normative development of auditory-language pathways and cerebral lateralization within the period of heightened neural plasticity. In contrast, postlingually deaf individuals typically have intact auditory pathway development and have established a robust linguistic foundation and auditory memory prior to hearing loss. Their post-CI rehabilitation therefore resembles a process of “activation” and “relearning,” rather than de novo “construction.” As a result, these patients generally exhibit faster auditory adaptation and superior initial performance compared to prelingually deaf peers [46]. Nevertheless, longitudinal studies suggest that the performance gap between the two groups tends to narrow over time [47].

The critical window for the DoD is defined primarily through studies on unilateral hearing loss. A systematic review indicates that longer periods of deafness, specifically congenital deafness lasting more than 4 years or peri-lingual deafness exceeding 7 years, are significantly associated with poorer auditory outcomes and reduced subjective benefit after cochlear implantation [48]. Long-term follow-up research further shows that children with congenital unilateral deafness attain the best speech perception results when implantation occurs before age 3 and when the duration of deafness is shorter than 3 years [n]. Likewise, in perilingual and postlingual unilateral deafness, the length of the deaf period remains an important predictor of rehabilitation outcomes, though a precise cutoff for post-lingual cases has not yet been established [43]. For most children with bilateral prelingual deafness, particularly those with congenital hearing loss, the age at implantation is approximately equal to or strongly correlated with the DoD. As a result, age at implantation is commonly used in research as a reliable and more precisely quantifiable metric for assessing the long-term effects of auditory deprivation. However, for individuals with bilateral perilingual or postlingual deafness, the specific critical threshold of the duration of deafness remains to be determined.

In summary, the onset of deafness determines the extent of pre-existing language and cognitive foundations, while DoD influences the degree of auditory pathway degeneration. For children with congenital or prelingual deafness, the cornerstone of successful outcomes is “early screening, early diagnosis, and early implantation”—to enable CI within the critical period of neural plasticity and thereby maximize the recovery of auditory and speech abilities. In contrast, for individuals with postlingual or acquired hearing loss, implantation should be promptly pursued following comprehensive candidacy evaluation.

### 3.2. Etiology of Hearing Loss

The etiology of hearing loss is a key predictor of CI outcomes in children. Different etiologies affect the inner ear structure, auditory nerve integrity, and auditory pathway development through distinct pathogenic mechanisms, potentially resulting in varying degrees of cochlear nerve dysfunction, cochlear malformations, or associated complications. These pathological changes can directly or indirectly influence the effectiveness of auditory and speech rehabilitation following CI.

#### 3.2.1. Genetic Etiologies

According to statistical data, more than 50% of neonatal SNHL cases are attributable to genetic factors [49]. The influence of specific gene mutations on CI outcomes is complex and variable, closely associated with the anatomical sites and distribution patterns of these mutations within the cochlea and the auditory pathway.

Deafness-causing genes predominantly expressed in the membranous labyrinth, such as GJB2 and OTOF, are generally associated with favorable outcomes following CI. The GJB2 gene, located on chromosome 13q12, encodes connexin 26—a protein widely distributed in supporting cells of the organ of Corti, spiral ligament, and cochlear fibrocytes [50]. Nishio et al. [51] demonstrated that individuals with genetic etiologies exhibit significantly superior performance in auditory skills, vocabulary development, and speech perception compared to those with structural abnormalities such as IEMs or CND. Subgroup analyses further revealed that among the genetic etiology group, patients with GJB2 and SLC26A4 gene mutations showed particularly favorable developmental trajectories [51]. Furthermore, Varga et al. [52] confirmed that deaf children with biallelic GJB2 mutations achieved significantly better outcomes across multiple auditory and speech assessments during long-term follow-up when compared to children with other etiological causes. It is important to recognize that the GJB2 mutation alone is not consistently an independent prognostic factor. The observed favorable prognosis associated with this mutation may be attributable to a constellation of advantageous clinical features commonly present in this patient population, including more typical cochlear involvement, fewer concomitant anomalies, and earlier initiation of intervention. Therefore, accurate assessment of individual prognosis should integrate both genetic genotype and detailed clinical phenotype to enable personalized evaluation and management.

The OTOF gene, located on chromosome 2p23, encodes otoferlin, a transmembrane protein highly concentrated in the basolateral region of inner hair cells and integral to presynaptic structure. Otoferlin also functions as a calcium sensor at ribbon synapses and plays a critical role in synaptic vesicle exocytosis [53]. Patients with OTOF mutations typically present with presynaptic auditory neuropathy or synaptopathy. Nearly all such individuals exhibit excellent sound perception and speech recognition following CI [54].

The SLC26A4 gene resides at chromosomal location 7q and encodes pendrin, a transmembrane anion transporter. Mutations in this gene can lead to non-syndromic hearing loss associated with enlarged vestibular aqueduct (EVA) or syndromic Pendred syndrome, which is characterized by SNHL, EVA, and thyroid dysfunction [55]. The association between SLC26A4 mutations and favorable outcomes following CI remains controversial. While several studies have reported excellent auditory performance in individuals with SLC26A4 mutations [56], others have found no significant difference compared to non-SLC26A4 cases [57].

In contrast, genes primarily expressed in spiral ganglion neurons—such as CHD7 and DDP1/TIMM8a—are linked to syndromic forms of hearing loss, and CI outcomes in these cases tend to be less favorable and more variable across individuals [58,59]. Furthermore, research has reported that pathogenic variants in the PCDH15 and DFNB59 genes are specifically associated with suboptimal outcomes following CI. This may be attributed to the involvement of the auditory nerve in PCDH15-related mutations, whereas DFNB59 variants are known to affect spiral ganglion neurons (SGNs) and brainstem auditory nuclei, potentially compromising central auditory pathway integrity [60].

#### 3.2.2. Status of Inner Ear and Auditory Nerve

The inner ear and auditory nerve, as critical components of the auditory pathway, are key determinants of rehabilitation outcomes following CI [61,62]. In cases of common IEMs, such as EVA, although there is a risk of perilymphatic fluid leakage during surgery [63], current evidence indicates that EVA is generally associated with favorable prognostic outcomes. A systematic review demonstrates that the overall effectiveness of CI in children with EVA is comparable to that observed in children with SNHL without IEMs [64]. Liu et al. [65] reported that children with LVAS exhibit more rapid development of preverbal auditory skills after CI compared to typically developing children. This advantage may stem from early residual hearing experience in infancy, which supports initial auditory learning and contributes to enhanced post-implantation progress [66].

Mondini dysplasia, a specific form of cochlear hypoplasia, is characterized by inherent anatomical abnormalities such as a reduced number of cochlear turns and diminished SGN population. Nevertheless, the residual functional SGNs are sufficient to support language discrimination ability [67]. Current clinical evidence demonstrates that the overall prognosis following CI is favorable, with most children achieving significant gains in auditory and speech development [65,67,68]. A 7-year longitudinal follow-up study revealed that the long-term auditory performance of children with Mondini dysplasia after CI is comparable to that of peers without inner ear malformations. However, when accompanied by bony cochlear nerve canal (BCNC) stenosis (<1.4 mm), word recognition scores are significantly lower. Importantly, this study further demonstrated that preoperative imaging assessment of BCNC dimensions and cochlear nerve integrity can effectively predict postoperative speech perception outcomes [69].

ANSD is a hearing disorder characterized by impaired neural transmission due to dysfunction at the level of inner hair cells, ribbon synapses, spiral ganglion neurons (SGNs), and/or the auditory nerve itself [70]. Based on the site of pathology, ANSD is categorized into a presynaptic type, where the lesion involves the inner hair cells or their synaptic junctions with the auditory nerve, and a postsynaptic type, which affects the spiral ganglion neurons or the auditory nerve distal to the hair cell-neuron synapse [71]. ANSD is primarily characterized by impaired subcortical neural synchrony, which may result from dysfunction of the auditory nerve, neuronal loss, or dyssynchronous neural firing. This disruption leads to an absent frequency-following response, reflecting a deficit in temporal auditory encoding [72]. As a result, individuals with ANSD exhibit significantly poorer speech recognition in noise than would be predicted by their pure-tone thresholds, despite often maintaining relatively intact speech perception in quiet environments [72]. A systematic review demonstrates that CI outcomes in children with ANSD are strongly associated with the site of neural dysfunction [71]. Children exhibiting presynaptic lesions, frequently linked to genetic mutations such as those in the OTOF gene, typically achieve superior postoperative auditory and speech perception outcomes compared to those with postsynaptic lesions, including conditions like auditory nerve dysplasia [73]. In contrast, the prognosis for children with postsynaptic involvement is more variable and often less favorable, with therapeutic benefits generally being more limited [71]. Lin et al. [73] demonstrated that CND is a significant contributor to poor outcomes following CI in both pediatric SNHL and ANSD patients. Their findings confirm that the presence and diameter of the cochlear nerve on preoperative imaging are critical determinants in the assessment of CI prognosis.

### 3.3. Preoperative Use Timing and Duration of HAs

For children with residual hearing undergoing CI, early preoperative fitting of HAs is associated with significantly improved postoperative auditory and speech rehabilitation outcomes, as well as enhanced quality of life, and demonstrates predictive value for long-term success [74]. This benefit may be attributed to the role of HAs as a form of preoperative auditory intervention, which supports the development of the auditory brainstem [75], mitigates maladaptive cross-modal cortical reorganization [76], and provides essential acoustic input during critical periods for language acquisition.

Continuous use of HAs prior to CI is generally associated with beneficial effects on auditory outcomes [77]. However, evidence indicates a nonlinear relationship between the duration of preoperative HAs use and the preservation of auditory cortex gray matter, with early HAs fitting showing a significant positive effect. Notably, this neuroprotective benefit begins to diminish beyond 17 months of use [78]. Furthermore, prolonged HAs use has been linked to poorer post-implantation performance in language comprehension, expressive abilities [79], and communication skills [80]. This adverse association may arise because extended reliance on acoustic amplification can delay the timely activation of the CI—the critical period for robust auditory input—potentially leading to restricted auditory experience and delayed speech development. Therefore, hearing-impaired individuals who do not meet CI criteria should be fitted with HAs to ensure sustained and developmentally appropriate auditory input to the central auditory system. In contrast, for patients whose hearing levels fulfill established CI candidacy requirements, HAs are unable to provide adequate auditory compensation. These individuals should receive CI without delay to prevent missing the optimal intervention window, thereby minimizing the risk of impaired auditory development and suboptimal postoperative outcomes.

## 4. Predictors Related to CI

### 4.1. Bilateral Implantation vs. Unilateral Implantation

When the clinical conditions of the patient permit, bilateral CI has become a standard medical practice. Compared with unilateral implantation, bilateral implantation offers binaural auditory advantages, including improved sound source localization, enhanced speech understanding in noisy environments, improved receptive and expressive language performance, and potentially more balanced auditory cortex development [81,82,83,84]. In clinical practice, bilateral implantation can be divided into simultaneous and sequential surgeries. In most countries, sequential implantation is still the main approach for bilateral CI users. Studies have confirmed that simultaneous implantation is superior to sequential implantation in terms of binaural auditory and speech rehabilitation outcomes, auditory brainstem pathway development, and surgical cost-effectiveness [85,86,87]. The prognosis of sequential implantation in the second ear may be influenced by the inter-implant interval. Evidence indicates that an interval exceeding 4 years significantly impairs speech understanding ability in the second ear, with longer intervals showing a significant negative correlation with speech test outcomes [88]. Hsu et al. [89] demonstrated that the duration between bilateral implantations is a key predictor of second-ear speech perception performance, particularly in noisy environments—longer intervals are associated with progressively poorer speech recognition in noise. Notably, when the interval exceeds approximately 9.6 years, speech recognition scores in noise for the second ear may fall below 30%, indicating suboptimal implantation outcomes. However, conflicting evidence exists; one study reported no significant association between speech perception improvement and either the inter-implant interval or the age at second implantation [90]. Therefore, further research is warranted to clarify the impact of the inter-implant interval on second-ear outcomes and to refine evidence-based guidelines for the optimal timing of sequential cochlear implantation.

### 4.2. Choice of Electrode Array and Electrode Position

The choice of electrode array and its intracochlear placement significantly influence postoperative auditory performance and speech rehabilitation outcomes. Clinical studies directly confirm the critical role of electrode position. For example, research by Alothman et al. [91] demonstrates that in prelingually deafened children, cochlear coverage by the implanted electrode array is a significant predictor of the Speech Discrimination Score (SDS); when coverage reaches ≥82.78%, children show significantly better speech discrimination ability compared to those with lower coverage. The study further finds that the FLEX 28 electrode array, which achieves deeper insertion (approximately 530° angular depth), provides greater cochlear coverage and better SDS improvement than the FORM 24 array (approximately 460° angular depth). This directly illustrates how different electrode array designs, by influencing final implant position, exert differential effects on auditory rehabilitation outcomes [91]. Fan et al. [92] found that greater electrode insertion angle is significantly correlated with better speech recognition performance: in the pediatric group, it positively correlates with monosyllabic word recognition rates at 6 and 12 months postoperatively. Their multiple regression analysis further confirms insertion angle as an important independent variable predicting bisyllabic word recognition in children after implantation. These findings align with the main conclusions of the systematic review by Breitsprecher et al. [93], which summarizes 23 studies and indicates that while most evidence supports a positive correlation between insertion depth and speech perception, the influence may vary depending on test materials (e.g., monosyllabic words vs. sentences), follow-up duration, and electrode type.

Furthermore, for bilaterally implanted children, symmetry in electrode insertion depth between the two ears is crucial. Research by Zavdy et al. [82] shows that if the angular insertion depth asymmetry between bilateral electrodes is ≥40 degrees, the risk of children requiring special education or support programs increases sixfold; the degree of asymmetry is also significantly higher in children in special education (55.7 ± 34.7 degrees) compared to those in mainstream education (29.5 ± 22.5 degrees). Simultaneously, greater insertion depth (aDOI ≥ 400 degrees) is significantly associated with better auditory performance (CAP ≥ 6). These findings strongly indicate that electrode array selection and precise intraoperative control of bilateral electrode position are fundamental surgical factors influencing auditory function reconstruction, binaural integration, and long-term educational outcomes in pediatric implant recipients.

## 5. Conclusions and Prospect

In summary, successful auditory rehabilitation following pediatric CI is shaped by a hierarchy of interdependent factors. Foremost among these is the timing of intervention, where earlier implantation and a shorter duration of deafness are paramount for harnessing developmental neuroplasticity. The child’s inherent capacities—such as stronger preoperative receptive language skills, speech recognition, higher developmental quotient, and nonverbal intelligence—constitute a critical foundation that modulates the effectiveness of auditory learning during this sensitive period. Concurrently, the familial context, particularly higher parental education levels, significantly influences the quality of postoperative support. From a clinical perspective, bilateral implantation and specific etiologies further refine prognostic expectations. Future research should focus on integrating these predictors into dynamic models to inform truly personalized rehabilitation strategies and support the full trajectory of auditory development—from basic sound detection to advanced speech comprehension.

## Data Availability

No new data were created or analysed in this study. Data sharing is not applicable to this article.

## Abbreviations

The following abbreviations are used in this manuscript:

CI	Cochlear implantation
SNHL	Sensorineural hearing loss
HAs	Hearing aids
ANSD	Auditory neuropathy spectrum disorder
CND	Cochlear nerve deficiency
IEMs	Inner ear malformations
EVA	Enlarged vestibular aqueduct syndrome
SDS	Speech discrimination scores
PLS-AC	Preschool Language Scales–Auditory Comprehension
CAP	Categories of Auditory Performance
IT-MAIS	Infant-Toddler Meaningful Auditory Integration Scale
MUSS	Meaningful Use of Speech Scale
SIR	Speech Intelligibility Rating
DQ	Developmental quotient
WISC-III	Wechsler Intelligence Scale for Children-Third Edition
DoD	Duration of deafness
SGNs	Spiral ganglion neurons
BCNC	Bony cochlear nerve canal
